# User involvement in the making: Positions and types of knowledge enacted in the interaction between service users and researchers in user panel meetings

**DOI:** 10.1111/hex.13281

**Published:** 2021-05-28

**Authors:** Per Koren Solvang, Unni Sveen, Helene Lundgaard Søberg

**Affiliations:** ^1^ Department of Physiotherapy Oslo Metropolitan University Oslo Norway; ^2^ Department of Occupational Therapy, Prosthetics and Orthotics Oslo Metropolitan University Oslo Norway; ^3^ Department of Physical Medicine & Rehabilitation Oslo University Hospital Oslo Norway

**Keywords:** health and social care research, panel meetings, patient and public involvement, positioning theory, types of knowledge, user involvement

## Abstract

**Background:**

Numerous studies of user involvement in research have been conducted. However, there is a lack of studies applying observational methods and addressing the concrete practice of involvement.

**Objective:**

To determine what knowledge types and competences users apply when involved in the research process through user panel meetings.

**Design:**

User panel meetings in a qualitative project in rehabilitation were sound‐recorded and transcribed verbatim. Data analysis applied an abductive approach framed by positioning theory.

**Setting and participants:**

Six rehabilitation service users and a similar number of researchers met 20 times during a six‐year project period. They discussed various issues in the research process such as interview guides, analysis and dissemination of results.

**Findings:**

The service users combined their respective knowledge and competence into six positions enacted in the panel interactions. They engaged as co‐researchers, based their contributions on their respective personal histories, represented an NGO and peers, applied their respective professional and educational backgrounds and, finally, engaged as concerned citizens.

**Discussion and conclusion:**

The findings add to the discussion of professionalization of user involvement by introducing a wider array of positions enacted than do the findings of previous studies. Researchers recruiting user panel members, as well as NGOs appointing candidates for user panels, are advised to consider a wide competence profile for possible candidates. A panel is also considered as a resource in confirming and elaborating on a study's findings.

**Patient and public contribution:**

A service user panel contributed to the study.

## 1 INTRODUCTION

The last 20 years have seen the increasing involvement of patients in steering health research. Grant applications are now expected to outline how people whose lives the proposed research will affect are to be involved.^1^, ^2^ Hence, increasingly, patients act as representatives providing their unique expert knowledge. Moreover, we are all potential patients and have the democratic right to a say in health research. This democratic role is framed by the concept of user involvement in research.

Numerous studies of user involvement in research have been conducted. One group of studies addresses the research process. A first question is in what way the participation is organized and what roles are taken by user participants. A guiding point has been a ladder of participation, where involvement is described on a scale from co‐option and consultation to collective action and co‐researching.^3^ A second question is the form of participation. In a recent literature review, Bird et al identified a broad set of ways in which the participation is organized. Users were members of steering committees and advisory boards, consultants, co‐designers and contributors to knowledge translation and took on specified roles in the research process, such as interviewers.^2^ A third question addresses the possible effect on the research process if user participation is applied. These studies typically identify the planning process and recruitment of participants as areas where user representatives have an impact.^4^, ^5^

Another group of studies addresses the users participating. One question is who is recruited to user participation. The studies addressing recruitment demonstrate that highly skilled, resourceful and strongly engaged people dominate in user involvement in research.^5^, ^6^, ^7^ A second question concerns the impact on users and their social status when users are included as co‐producers of research. Their participation is judged to be an empowering process stimulating further activism on behalf of the group they represent.^8^ A third question addresses how users participate in the research process. Here, a key issue is the possible professionalization of users because they often turn into scientifically engaged lay experts.^7^

In the critical disability movement and in mental health research, epistemological challenges to user involvement in research have been formulated. In an early contribution, Mike Oliver distinguished between positivist and interpretative science on the one hand and emancipatory science on the other. The latter is based on how disabled people formulate their interests in order to change society and fight ableism.^9^ A recent contribution to emancipatory disability studies highlights the importance of disabled people playing an active, paid role in knowledge production and in the case of critical autism studies ‘ensuring there is no sustainable dichotomy between autistic and non‐autistic authorship’.^10^ Psychiatric research is another arena where epistemological issues have been addressed. It is asserted by the psychiatric survivor movement that they have not fought to have a voice in research only to improve the research process as helping hands. Given the history of oppression in psychiatry, the power structures within the discipline need to be altered.^11^, ^12^, ^13^ The current study does not fundamentally challenge the power relations in rehabilitation research but contributes to the recognition of competence by analysing the wide scope of knowledge enacted in user involvement.

In the studies of how users participate, two lacunae can be observed. First, what do user participants contribute when involved in the research process? This is a question seldom addressed in detail. Second, studies based on observational data are scarce. The current study will contribute to lessen this knowledge gap. A user panel has been followed over time by making audio recordings of the meetings where both users and researchers participate. The leading question for analysing the panel interaction is what types of knowledge and competences users apply when they influence the research process through panel meetings.

The analysis of the interaction tries to shed light on three interrelated questions. First, which positions are brought to the fore by user participants in their contributions to the panel work? Second, what knowledge types and competences do participants draw on in the user–panel interactions. The third question addresses how the interaction between users and researchers contributes to the roles and types of knowledge realized by users in panel meetings. All questions address role behaviour and will be well facilitated by a theoretical framework that engages with positions taken in social interaction.

## 2 POSITIONING THEORY

The position of selves is not static, but a dynamic process taking place in encounters between social actors. In a seminal paper, Davies and Harré outline a perspective where a concept of positions replaces the role concept. When people meet and interact, positions are created.^14^ A key point is that social actors both relate to the ostensible topic at hand when meeting and bring in a diverse set of elements from their biographies: ‘In speaking and acting from a position people are bringing to the particular situation their history as a subjective being, that is the history of one who has been in multiple positions and engaged in different forms of discourse’.^14^ What goes on is a form of prepositioning whereby people bring their skills, character traits and personal biographical patterns of experience to the situation at hand.^15^

In a discursive practice, of which the series of user panel meetings is an example, subject positions are made available to participants. These positions are built on both the conceptual repertoire a person possesses and the structure of rights to use this repertoire. The panel meeting as a discursive practice is such a structure that actualizes specific concepts, experiences and story lines as meaningful when contributing to the discourse. Specific speakers are positioned by the ideas they bring into the conversation about their own positions. In addition, the conversation itself will contribute to the positioning of the specific speaker by the other participants and their ideas about that speaker.^15^ In a user panel, participants will typically scan their pasts for occasions similar to the panel meeting so as to pick up hints about what is expected of them. The positioning of lay members of the panel will be further moulded by how a participant is understood by the other discourse participants, in this case other lay members and the researchers.

## 3 METHOD

The interdisciplinary research project Transitions in Rehabilitation, carried out at Oslo Metropolitan University in the years 2013‐2019, established a user panel that met 3‐4 times a year.^16^ The study was planned to end in 2017 but the project period was extended because of two maternal leaves and one case of long‐term illness among the PhD candidates contributing to the study. The project was a qualitative study of accident‐injured people suffering from traumatic brain injury and multitrauma, and the professionals facilitating the rehabilitation process of the accident‐injured, both in specialized hospitals and in services at the municipal level. The leading research question was how injured individuals and professionals perceived the transition from specialized hospital‐based rehabilitation to everyday life in family, employment and local community. The user panel comprised both people injured themselves and people caring for injured children. Two disability nongovernmental organizations (NGOs) were contacted and asked to recruit panel members. Six panel members were recruited. They met with researchers involved in the project in 20 meetings during the 6‐year project period. An overview of panel participants is given in Table 1.

**TABLE 1:** Participants in user panel meetings

ID code	Age group in 2016	Gender	Education	Occupation	Injury experience	No. of meetings
User 1	60‐70	F	Teacher education (bachelor)	Disability NGO^a^/disability pension	Multitrauma with TBI^b^	15
U2	50‐60	M	Accounting	Accountant/disability pension	TBI	16
U3	30‐40	M	Finance	Bank employee	Spinal cord injury	13
U4	40‐50	M	Master's political science	Employee in governmental institution	Spinal cord injury	8
U5	50‐60	F	Occupational therapy (bachelor)	Occupational therapist in municipal services	Multitrauma with TBI‐injured child	18
U6	50‐60	F	Not revealed	Not revealed	Multitrauma with TBI‐injured child	13
Researcher 1	50‐60	F	PhD sociology	Professor and project head	None	18
R2	30‐40	F	PhD social anthropology	Project coordinator	None	10
R3	50‐60	M	PhD sociology	Professor	None	13
R4	50‐60		PhD health sciences	Physiotherapist and associate professor	None	14
R5	50‐60	F	PhD health sciences	Occupational therapist and professor	None	12
R6	60‐70	F	PhD health sciences	Associate professor	None	7
R7	60‐70	F	PhD medical anthropology	Head nurse and associate professor	None	11
R8	40‐50	M	PhD sociology	Associate professor	None	4
R9	30‐40	F	Medical doctor	PhD candidate	None	5
R10	30‐40	M	Master's organization studies	PhD candidate	TBI	6
R11	30‐40	F	Master's sociology	PhD candidate	None	3

^a^ Nongovernmental organization.

^b^ Traumatic brain injury.

Panel meetings occurred in the late afternoons in a meeting room at the Oslo Metropolitan University's downtown campus. The meetings were led by the project head and lasted for three hours. On the agenda were issues such as interview guides, recruitment strategies, presentations of drafts for analysis, the basics of the qualitative method, dissemination of results, needs for further research and evaluations of the panel's work. The number of participants in each meeting varied. Typically, a meeting would consist of 5‐10 people, where at least half of the participants were users. An agenda was set up two weeks prior to the meetings and research materials such as interview guides, selected interview transcripts, abstracts for scholarly papers and drafts for dissemination texts were attached. The structure of the meetings was presentations by researchers that users responded to and discussed among themselves and with the researchers. Most panel meetings were, in agreement with the participants’ informed consent, sound‐recorded and transcribed verbatim. Three meetings were documented by minutes only.

The data analysis followed the spiral approach outlined by Cresswell.^17^ All authors read the transcripts. The readings served both as a reminder of what occurred in the meetings the authors attended and as a detailed representation of what occurred in the meetings they did not attend. Based on both the literature and what emerged in the meetings, the first author made a list of possible positions and types of knowledge relevant when practising user participation in research.^18^ The authors worked further with classifying and describing positions spoken from and types of knowledge applied by user representatives. The authors met and compared their classifications and discussed questions that emerged in the coding process. This process was summed up by the first author by making a summary of each meeting, paying close attention to positions and knowledge types applied, as well as to identifying illustrative passages. Using the series of analytically framed meeting summaries, the first author drafted the article. All authors revised the early drafts and the first author completed the final draft. The final version was approved by all authors.

In the analysis, we applied an abductive approach. Unlike induction, abduction begins with data derived from a theory‐informed research agenda, but unlike deductive analyses, abductive analyses are not bound by hypotheses formulated before the production of data.^19^ Hence, abduction is a creative process aimed at producing knowledge and new hypotheses from unanticipated empirical observations.^20^ Our unanticipated empirical observation was that lay panel members actively used their competences as professionals. We used positioning theory to organize this observation into a framework for further analysing the social interaction that occurred in the meetings.

The authors of this article are identified as R3, R4 and R5 in Table 1. Because we participated in the meetings, our analysis has an element of reflection on the process(es) in which we participated. The idea to study positions and types of knowledge enacted emerged from our participation in the meetings. To ensure transparency, we have strictly kept to information represented in written transcripts of the meetings. Additionally, the abductive approach adds to the transparency of the analyses. The development of Table 2 was based on using positioning theory as a lens for reading the transcripts. In this development, we have used a grid that constitutes a point of reference outside our strictly personal experiences of the meetings. The user panel was in one of the final meetings introduced to the findings section of the current article and provided valuable comments and reflections. Involvement at the level of co‐authorship by panel members was discussed but declined by the panel members, in part because they did not see this type of involvement as part of their assignment as panel contributors. Also, they were reluctant to engage themselves in producing a foreign language paper.

**TABLE 2:** Overview of positions and types of knowledge enacted in user panel meetings

Knowledge gained from	Positions enacted
Personal history about experiences as service user relations to family and friends impairment experience participating in work life and in the public arena	Co‐researcher
	Affected individual
	Disability NGO representative
	Professional
	Concerned citizen

Peer interaction	
NGO^a^ engagement	
Positions as user representatives	
Education	
Professional work experience	
Broad citizen engagement	

^a^ Nongovernmental organization.

This study was evaluated by the Data Protection Official at the Norwegian Centre for Research Data. Both researchers and users gave their informed consent to participate in the study. All participants were made aware that they could withdraw from participation at any time during the analysis without affecting their involvement as researchers or panel members in the project in general. Participants agreed to take part even though anonymization could not be fully guaranteed, because it was known in advance who participated in the project. Confidentiality and respect for each participant were emphasized.

## 4 FINDINGS

The user representatives enacted five social positions and based their engagement and contributions on six broad types of knowledge. Concerning knowledge learned from their personal histories, this knowledge can be divided into three aspects. These positions and types of knowledge are outlined in Table 2.

The positions and the knowledge types emerged in closely interrelated ways, and they were facilitated by the interactional patterns in the panel meetings. The presentation of findings will be organized around the five positions enacted.

### 4.1 The co‐researcher

User participants were asked by researchers to assist in three main phases of the research process. First, they gave advice about issues of vulnerability in the empirical project phase. Users pointed out the importance of speaking with interviewees in all stages of coming to terms with the consequences of the accident, even if interviewees were considered by staff to be in a vulnerable phase. Second, users were introduced to drafts of scientific papers in various stages of completion. Here, they contributed reflections that deepened interpretations suggested by researchers. The papers presented were based on interviews with both injured individuals and professionals involved in the traumatic brain injury (TBI) and multitrauma rehabilitation processes. At an early stage in the series of user panel meetings, an introduction to qualitative methods was agreed upon. The initiative came from the user representatives, and in meeting 5, R6 and R7 carried out a teaching session on qualitative methods.

Users differed in their approaches to the analytical process. U4 held a master's degree in social science and frequently contributed analytical perspectives based on his educational background and his intimate knowledge of the logics of research in the social sciences. In analysing professional work, the other users frequently were opposed to the analytical language applied. Below, R3 is referring to statements in a previous discussion about using analytical language.

U3: You are saying that one must write two versions of the same article. One for the scientific journals read by other researchers, where you are bound by certain requirements, as you say. But if it is to be published in a popular science journal of some kind, you will have to rewrite, to change some of the most distancing words and sentences.

U6: That is what we have been talking about, using researcher language, and the use of colloquial language that we are able to understand. How important it is. But of course, if you are going to publish in a ….

U5: [Interrupts] Why must it be this kind of language in these scientific articles? I do not understand it. ‘Externalising’, I still do not understand what it means. Although you did explain it just a moment ago, I still do not understand.

R3: [Laughs] No, it is….

U5: So, whom…. what is the goal? Who is expected to understand this goal? What is it…? What does it bring?

The initial reflections by U3 echoed a previous response from the project head. She emphasized the importance of analytical language for studies in the social sciences. Otherwise, they would not be published. Using simpler language was to her important for dissemination only. U5 did not agree on this division and challenged the practice of social science publishing in a form that is difficult to access for the average educated citizen. In subsequent meetings, researchers held to their preferred division of labour, specialized language for scientific publication and simpler language for dissemination to the general public.

Dissemination was the third phase in the research process where user representatives were expected to contribute. Researchers expressed an expectation for the project results to be presented in journals published by the NGOs. This expectation was a frequent issue in the panel meetings, but such presentations of the results never seemed to materialize. On their side, users asked researchers if they ‘write in the newspapers’. The answer from the researchers to this question was that the type of research conducted by the project seldom gains interest in the press.

### 4.2 The affected individual

In panel meeting number 15, U6 pointed out to the researchers that ‘you are good at your job, and we know what we have, our own stories’. All panel members contributed to the discussions with perspectives grounded in their own personal histories. These discussions concerned four themes. The stories told dealt with the impairment experience, the relation to family, the service‐user position and the changes in personal identity when facing the public arena post‐injury. In addition, peer relations were brought into the mix.

It turned out that presenting users’ personal narratives was a success in contributing to disseminating research results. At the end of the project period, a public seminar was organized. It was well attended. Results from both the transitions project and collaborating projects in Denmark were presented. As part of the seminar, U2 and U6 were interviewed by R3. Users presented their personal stories and opinions on the health and social welfare services received and R3 provided scholarly frameworks for the narrations. In the final panel meeting, the huge success of the seminar interview was the talk of the day. Both the Danish guests and audience members were said to be thrilled by this part of the seminar.

A frequent way of contributing to panel discussions was by sharing an opinion that generalizes based on the participants’ personal experiences. Here, R11 had introduced the issue of loneliness in analysing interviews of accident‐injured people. U2 had a TBI and struggled with cognitive deficits:

U2: You can be lonely even if you have family and friends. In some way, you are alone even if you are together with other people, or if not. It is not so easy to do anything about that. That loneliness you must manage in some way. That is not so easy to understand for those who are well. If you also happen to *look* [U2’s emphasis] healthy, that only worsens it. Then you are expected to be as you were before the injury. You don’t look ill, but your brain does not work as it did. Loneliness has something to do with these things.

The impairment has driven him into a kind of limbo between the disabled and non‐disabled position that affects how he understands himself. He shared this change of identity as a contribution to the discussion of how loneliness is an element in accident‐injury recovery.

Impairment came into panel discussions very concretely. U1 and U2 lived with the cognitive consequences of TBI. They asked for simple language, enough time to read material before meetings, and they even made revisions to their own comments because of the possibility of poor understanding of what was going on in meetings. Impairment also came into the discussions when participants commented on findings from the patient interviews.

U3: I have used a wheelchair for 25 years. I do not think about it any longer, that I do not walk. I am so used to it. But there are many other things: One example is not controlling bladder and bowel function as you did before. Skin sensation is also difficult. It is easy to get ulcers if you have no skin sensation.

By this sharing, U3 elaborated on the preliminary finding that bladder and bowel functions are silenced in public discourse on disability, in favour of wheelchair accessibility.

The intimate relation between personal experience and peer relations came through in the frequent discussions and evaluations of user participation.

U6: When we have such a long experience, we have been through many other situations, with other users. In effect, I think we speak in general, not only out of our own experience, I think, but also for those who are not able to stand up. I think we put a word in for them as well.

U3: I agree. If we on the panel can bring with us our flock, you know, see the whole situation and describe it, not only of our own situations, but we have seen many. That is, all those I have met through time and seen the different consequences for them, you know, how people react and things like that. I can take this with me and present it, not think out of my own head only.

Such reflections were instigated by both the evaluations of the participation process and the fact that the participation was to be studied. The relations between the personal and representing peers were of course actualized by the fact that user participants were assigned to the panel by the NGO where they were active members.

### 4.3 Disability NGO representative

U1 is the one who most explicitly brings in perspectives and priorities from the NGO. She works part time for the NGO from which she, U3, U5 and U6 were recruited. Some issues she discusses with others working at the NGO’s headquarters even prior to the panel meetings. In an evaluation, U1 reflects actively on her twin positions:

U1: Regarding being a user representative, I have tried to think: ‘Not only me, but all those people I have talked to through the years.’ Represent them well. I don’t know how well I have accomplished it, but I have at least tried to have it in mind. I have also brought things [from the panel meetings] and discussed them in the organisation. I have wanted to make sure that it was not only my opinion I have been voicing but [to make sure that I] talk it through with others.

At an early stage in the project's course, she initiates a scheduled short meeting of user representatives prior to panel meetings with researchers. This short meeting takes place in the same locality as the subsequent panel meeting half an hour before researchers arrive. U1 wanted to establish the users as a distinct group having their own forum as well as having a specific forum for researchers. The pre‐meetings were not sound‐recorded, and they occurred only in the middle phase of the series of panel meetings.

### 4.4 The professional

The study design included interviews with both professionals and injured individuals. When recruiting user representatives, only the positions as an impaired person or as a relative of an impaired person were considered legitimate types of experience. What occurred was that users also spoke of their experiences as professionals. This practice was most clearly expressed by U5. In one of the first panel meetings, she asked for permission to share from her professional experience as an occupational therapist. The project head gave U5 permission to do so. In later meetings, she actively contributed her experiences from this position.

U5: There are a lot of challenges. I am thinking about my daughter, who was injured 20 years ago and the long process ending up with today. I have felt that I really needed the competence I have as an occupational therapist. Without it, I would not have been able to manage. Today, I work in the municipal health services and meet many parents with kids and youngsters with various injuries. It is not easy. There are very, very big challenges and the kids’ and youngsters’ situations are heavily dependent on the parents’ efforts. I do not know how big the topic of parents is in rehabilitation. I think it would be suitable for study.

U5 was not unique. U1 frequently referred to her practice as a teacher prior to suffering her injury. The other users also, in various ways, brought in their professional work experience. U3 referred to disability inclusion when he was a mid‐level manager and U4 frequently based his comments on perspectives drawn from his knowledge of social theory after completing his social science degree.

Three researchers in the project had combined positions. They worked part time as associate professors at the university and part time as professionals at two hospitals, as nurse, occupational therapist and physiotherapist, respectively. They frequently drew on their professional experience and shared perspectives from client work. This sharing was not reflected on but was understood as an integral part of their competence as participants in an interdisciplinary research project. The researchers’ use of their positions as professionals contributed to frequent sharing of professionals’ perspectives in the panel meetings. This sharing was made relevant both because two‐thirds of the empirical material of the study were derived from interviews with professionals and because injured individuals spoke at length about their experiences with professional services.

### 4.5 The concerned citizen

The panel contributed to a project that concerned the organization of health services, an issue of great political significance. In the analysis of injured peoples’ narratives, issues such as family relations and gender roles were addressed. These themes of interest are politically significant as well. Hence, there was a framework of current issues present in panel discussions. Frequently, a position as a concerned citizen was enacted. One example was to emphasize what was in the benefit of society:

U6: There is a socioeconomic saving in providing good rehabilitation both for individuals and for the whole family. I think there is a lot of money to be saved by cooperating well and by not causing frustration for either users or their next of kin.

Another example was when gender as an analytical concept was introduced in a draft by R11 (who was not present in the panel meeting).

U5: I do not find it well motivated what she has presented. I do not find it valid. Men and women are different. We have different roles. We know that [laughs]. We are very different. That is not surprising. These are the thoughts I have.

Here, U5 refers to her belief that gender role differences are manifest and legitimate. This belief leads her to problematize the critical analysis of gender roles determining how the injured prioritize in their rehabilitation processes.

### 4.6 Distribution of positions

The various positions outlined were not evenly distributed among users. As already indicated, U1 most clearly formulated the NGO position, U4 the co‐researcher position, U5 the professional position, U6 the engaged citizen position and U2 and U3 the most engaged use of their personal stories in order to elaborate and comment on findings.

## 5 DISCUSSION

Our objective in analysing the panel meetings has been to better understand the positions enacted and the types of knowledge enacted by user representatives. The analysis has been thematically organized into five positions. First, users contributed as co‐researchers. They gave direct advice on design issues, engaged in learning qualitative methods and applied social science knowledge in discussing preliminary results. Second, users used their individual stories in contributing to all stages in the research process. They conveyed service‐user experiences and impairment experiences as well as stories about altered family relations. Third, they represented the NGO and knowledge deriving from both peer relations and previous experience from acting as user representatives. Fourth, their professional positions came into play. The two users with professional backgrounds in the health and educational services, respectively, used most extensively experiential knowledge from their positions as professionals. Finally, a number of contributions in the meetings derived from users’ positions as concerned citizens. Here, they raised issues such a priority setting priorities in health policies and how gender relations should be understood. Together, the five positions comprise the broad variety of positions and types of knowledge enacted in user participation in research.

Previous studies have addressed the possible professionalization of user representatives. In the process of professionalization, lay knowledge is in decline and other types of knowledge represented by users are on the rise. One example is the hybrid position of claiming both certified professional expertise and lay expertise.^7^ The current study adds nuance to the issue of professionalization. On the one hand, it demonstrates how higher education, professional practice experience and learning about scientific methods play a significant role. Users contributed to the discussions with arguments based on social science, engagement in NGOs and competences deriving from professional work in health. Thereby, the participation in the user panel is part of a broader professional competence. On the other hand, the lay perspective is certainly present. Users challenge the analytic language used by researchers, even in scholarly publishing. Regarding research dissemination, what gains momentum is the narration of personal histories as an integral part of the dissemination, exemplified by the seminar held in the final part of project period. In sum, the users demonstrate a version of hybrid expertise. Contributions they make as user representatives are derived from positions situated in different parts of their respective biographies, not only the disability experience and NGO engagement.^14^

What the current study adds to the literature is that it identifies the wide set of positions that constitute hybrid expertise. Hybridity is not only about personal experience and professional competence. It includes perspectives from NGO engagement and peer relations as well as a broad citizen engagement. Into the mix comes also educational background that gives form to a co‐researcher position in the analytical process. In sum, the findings demonstrate how the hybrid user position contains a complex set of knowledge types and social positions.

It should also be noted that to be a health professional is a mixed blessing when acting as a user representative. In a medical sociology perspective, it is a strength that both professional work and the client experience are represented. In a critical disability perspective, health professionals are frequently considered as representing oppressive and paternalistic discourses.^21^ From such a perspective, the position of a health professional as user representative could be considered unwise. In the user panel studied here, an active reflection on positions contributed to a transparency whereby all participants became aware of the mixed positions present in the panel meetings. Hence, there is a need for reflection among both researchers and service‐user representatives about positions in the recruitment process and when user participation unfolds.

Most studies on user participation do not question the usefulness of participation, but a few critically engage with the possible downsides. Malterud and Elvbakken have undertaken a review study on users as co‐researchers.^22^ They concluded that this type of participation gains too much attention at the cost of research quality. Forbat and Hubbard concluded that users as co‐researchers conducting interviews and introducing their own experiences changed the topic of conversation in a way that was recognized as counterproductive to the research interview as information gathering.^23^ Another challenge is the possibility that research designs will be forced in directions that favour the interests of specific patient groups.^24^ The current study adds to this critical engagement by indicating a need for reflection on users’ contributions to the analytical process. In the findings section, objections to the use of analytical concepts were applied and normative reluctance towards gender perspectives was demonstrated. On the one hand, there seems to be a danger that theoretical strengths of high‐level studies can be jeopardized when analytical processes must be shared with lay people. This point is in line with the critical discussion of member checking by Varpio et al^18^ On the other hand, such tensions can sharpen the way researchers ground and formulate their analyses. The result could be sound improvements in deciding what perspectives are to be chosen for analysis and the level of precision when they are formulated.

A final element to consider is a panel's potential role in providing additional focus group material for the project it serves and in contributing to research quality as defined by standards of qualitative methods. Discussions in the panel contained a high proportion of sharing experiences concerning subjects introduced by researchers. This was a process akin to what is gained from the focus group method.^25^ As demonstrated in the findings, one example was the panel's expanding on the importance of bowel and bladder functioning to disability discourse. These reflexions could have supported the analysis as additional empirical material but were difficult to include in the paper to be published on the experiences of the injured. A methodological requirement in qualitative research design is member checking, meaning that one or more representatives of the studied group are introduced to the analysis and contribute to validating results.^17^ Such a procedure occurred in the panel meetings. An added value was the professional backgrounds in health and educational services held by two user participants. They could contribute to member checking of the analysis of professional work from their positions as service providers. Based on these characteristics of the panel discussions, projects appointing user panels could consider the possibility of more actively including panel discussions as part of the methodological design.

### 5.1 Limitations

The authors of this study participated in the social interactions that were analysed. Precautions were taken to ensure rigour in the analytical process as outlined in the methods section. However, an independent analysis of the transcripts would have produced analyses with a foothold outside the views of user participation held by the authors. Another limitation concerns diversity in the panel. The members were predominantly white and middle class. Their homogenous social backgrounds and professional careers might have restricted the scope of inputs to the research process. However, some panel members had stopped working because of the consequences of their respective accidents and the panel discussions often contained reflections on the interests of accident‐injured people in more vulnerable positions than the panel members themselves were.

## 6 CONCLUSION

This study demonstrates the wide range of social positions and types of knowledge used by service users in a panel organized by a qualitative interdisciplinary research project in rehabilitation. Such a variety of competences is hinted at in other studies as well, but not systematized as in the current study. The systematization of positions is well suited to advise researchers recruiting user panel members as well as NGOs appointing candidates for user panels. The influence on the research process will not be based only on patient experiences and priorities set by a recruiting NGO, but also on the professional competences and educational training held by participants, as well as engagement in social issues on a broad scale.

## 7 CONFLICT INTERESTS

The authors declare that they have no conflicting interests to disclose.

## Data Availability

The data that support the findings of this study are available upon reasonable request from the corresponding author. The data are not publicly available due to privacy restrictions.

## References

[1] Green G. Power to the people: to what extent has public involvement in applied health research achieved this? Res Involv Engagem. 2016;2(1):1‐13. 29507763 10.1186/s40900-016-0042-y PMC5831888

[2] Bird M, Ouellette C, Whitmore C, et al. Preparing for patient partnership: a scoping review of patient partner engagement and evaluation in research. Health Expect. 2020;23(3):523‐539. 32157777 10.1111/hex.13040 PMC7321722

[3] Harris J, Cook T, Gibbs L, et al. Searching for the impact of participation in health and health research: challenges and methods. Biomed Res Int. 2018;2018:1‐12. 10.1155/2018/9427452 PMC5971326 29862298

[4] Brett J, Staniszewska S, Mockford C, et al. Mapping the impact of patient and public involvement on health and social care research: a systematic review. Health Expect. 2014;17(5):637‐650. 22809132 10.1111/j.1369-7625.2012.00795.x PMC5060910

[5] Domecq JP, Prutsky G, Elraiyah T, et al. Patient engagement in research: a systematic review. BMC Health Serv Res. 2014;14(1):1‐9. 24568690 10.1186/1472-6963-14-89 PMC3938901

[6] Boote J, Telford R, Cooper C. Consumer involvement in health research: a review and research agenda. Health Policy. 2002;61(2):213‐236. 12088893 10.1016/s0168-8510(01)00214-7

[7] Thompson J, Barber R, Ward PR, et al. Health researchers’ attitudes towards public involvement in health research. Health Expect. 2009;12(2):209‐220. 19392833 10.1111/j.1369-7625.2009.00532.x PMC5060486

[8] Reynolds J, Beresford R. “An active, productive life”: narratives of, and through, participation in public and patient involvement in health research. Qual Health Res. 2020;30(14):2265‐2277. 10.1177/1049732320961053 33012242 PMC7649936

[9] Oliver M. Changing the social relations of research production? Disabil Handicap Soc. 1992;7(2):101‐114.

[10] Woods R, Milton D, Arnold L, Graby S. Redefining critical autism studies: a more inclusive interpretation. Disabil Soc. 2018;33(6):974‐979.

[11] Carr S. ‘I am not your nutter’: a personal reflection on commodification and comradeship in service user and survivor research. Disabil Soc. 2019;34(7–8):1140‐1153.

[12] Friesen P, Lignou S, Sheehan M, Singh I. Measuring the impact of participatory research in psychiatry: how the search for epistemic justifications obscures ethical considerations. Health Expect. 2019;1‐8. 10.1111/hex.12988 31854081 PMC8137501

[13] Rose D. Service user/survivor‐led research in mental health: epistemological possibilities. Disabil Soc. 2017;32(6):773‐789.

[14] Davies B, Harré R. Positioning: the discursive production of selves. J Theory Soc Behav. 1990;20(1):43‐63.

[15] Harré R, Moghaddam FM, Cairnie TP, Rothbart D, Sabat SR. Recent advances in positioning theory. Theory Psychol. 2009;19(1):5‐31.

[16] Romsland GI, Milosavljevic KL, Andreassen TA. Facilitating non‐tokenistic user involvement in research. Res Involv Engagem. 2019;5(1):18. 31183162 10.1186/s40900-019-0153-3 PMC6551868

[17] Creswell JW, Poth CN. Qualitative inquiry and research design: Choosing among five approaches. Thousand Oaks, CA: Sage publications; 2016.

[18] Varpio L, Ajjawi R, Monrouxe LV, O'Brien BC, Rees CE. Shedding the cobra effect: problematising thematic emergence, triangulation, saturation and member checking. Med Edu. 2017;51(1):40‐50. 10.1111/medu.13124 27981658

[19] Patton MQ. Qualitative Research & Evaluation Methods, 4th edn. Thousand Oaks, CA: SAGE; 2015.

[20] Timmermans S, Tavory I. Theory construction in qualitative research: from grounded theory to abductive analysis. Soc Theor. 2012;30(3):167‐186.

[21] Goodley D. Disability Studies: An Interdisciplinary Introduction, 2nd edn. London, UK: Sage; 2017.

[22] Malterud K, Elvbakken KT. Patients participating as co‐researchers in health research: a systematic review of outcomes and experiences. Scand J Public Health. 2020;48(6):617‐628. 31319762 10.1177/1403494819863514

[23] Forbat L, Hubbard G. Service user involvement in research may lead to contrary rather than collaborative accounts: findings from a qualitative palliative care study. J Adv Nurs. 2016;72(4):759‐769. 26689175 10.1111/jan.12865

[24] Gibson A, Britten N, Lynch J. Theoretical directions for an emancipatory concept of patient and public involvement. Health (London). 2012;16(5):531‐547. 22535648 10.1177/1363459312438563

[25] Morgan DL. Focus groups and social interaction. In: Gubrium J, Holstein J, Marvasti A, McKinney K, eds. The Sage Handbook of Interview Research: The Complexity of the Craft, 2nd edn. Thousand Oaks, CA: SAGE; 2012.

